# High gravity and high cell density mitigate some of the fermentation inhibitory effects of softwood hydrolysates

**DOI:** 10.1186/2191-0855-3-15

**Published:** 2013-02-14

**Authors:** Nuwan Sella Kapu, Maya Piddocke, Jack (John) N Saddler

**Affiliations:** 1 Forest Products Biotechnology/Bioenergy Group, Faculty of Forestry, University of British Columbia, 2424 Main Mall, Vancouver, BC, V6T 1Z4, Canada

**Keywords:** High gravity fermentation, High cell density, Fermentation inhibitors, Steam pretreatment, Softwood, Yeast

## Abstract

After steam pretreatment of lignocellulosic substrates the fermentation of the biomass derived sugars to ethanol is typically problematic because of both the generally low sugar concentrations that can be supplied and the presence of naturally occurring and process derived inhibitors. As the majority of the inhibitory materials are usually associated with the hemicellulose rich, water soluble component, this fraction was supplemented with glucose to simulate high solids, un-detoxified substrate to see if a high gravity/high cell consistency approach might better cope with inhibition. Several yeast strains were assessed, with the Tembec T1, T2 and Lallemand LYCC 6469 strains showing the greatest ethanol productivity and yield. The addition of supplemental glucose enabled the faster and quantitatively higher removal of hydroxymethylfurfural (HMF). High cell density could provide effective fermentation at high sugar concentrations while enhancing inhibitor reduction. A 77% ethanol yield could be achieved using strain LYCC 6469 after 48 h at high cell density. It was apparent that a high cell density approach improved ethanol production by all of the evaluated yeast strains.

## Introduction

Biomass-based ethanol has the potential to provide substantial environmental, energy security and geopolitical benefits in comparison to fossil based transportation fuels. Over the past 30 years or so, ethanol from sugar and starch feedstocks has developed into mature industries which account for an annual global production of more than 80 billion liters of ethanol (Walker 2011). However, the “food vs. fuel” debate over the use of sugar and starch feedstocks has resulted in unfavorable public perception of ethanol production from these raw materials. Consequently, the potential of biomass-to-ethanol processes that can use non-food, sustainably renewed resources, abundant enough to have a significant impact on petroleum consumption, has attracted substantial scientific, political and commercial interest (Perlack et al. 2005, US Department of Energy 2011).

Commercialization of the lignocellulosic based ethanol process will require the cost-effective conversion of at least 80% of the original polysaccharides present in the feedstock to ethanol (Boussaid et al. 1999, Humbird et al. 2011). This dictates that each step of the overall process including the pretreatment, enzymatic hydrolysis, fermentation, and ethanol recovery steps needs to be optimized to achieve high ethanol yields. From a fermentation perspective, process optimization requires the successful mitigation of several process challenges. One of the biggest challenges encountered in a typical biomass-to-ethanol process is the low sugar concentrations that can be achieved after the pretreatment and hydrolysis steps of the process (Galbe and Zacchi 2002, Robinson et al. 2003, Galbe and Zacchi 2007, Sánchez and Cardona 2008, Humbird et al. 2011). Low concentrations of fermentable sugars invariably leads to low ethanol concentrations and, despite progress being made in increasing the efficiency of the “ethanol concentrating stage”, starting ethanol concentrations of greater than 4% w/v still help make product recovery more cost-effective.

A second challenge with trying to ferment biomass derived sugars is the presence and production of inhibitors which can cause the slowing or cessation of microbial cell growth, reduced ethanol productivity and low ethanol yield (Palmqvist and Hahn-Hägerdal 2000, Klinke et al. 2004, Almeida et al. 2007, Almeida et al. 2011). Inhibitors can be broadly categorized into those compounds the plant produces itself to restrict microbial growth and process-derived compounds (Boussaid et al. 2001, Liu 2010). Natural inhibitors are produced by plants as a defense against pests and pathogens while the process-derived compounds are generated during the pretreatment, fractionation and hydrolysis steps of the biomass-to-ethanol process. Many pretreatment processes can lead to the formation of a range of inhibitory material including furans (furfural, 5-hydroxymethyl furfural- HMF), phenolics and organic acids (Almeida et al. 2007). Furans are known to directly inhibit enzymes such as alcohol dehydrogenase, pyruvate dehydrogenase and hexokinase causing reduced ATP synthesis, and DNA damage has also been observed (Banerjee et al. 1981, Modig et al. 2002, Almeida et al. 2009, Liu 2011). Phenolics can affect cell membrane integrity resulting in the disruption of sugar transport and cell growth (Heipieper et al. 1994, Pienkos and Zhang 2009) while aliphatic acids have been shown to inhibit cell growth through the increased ATP drain needed to maintain cellular pH (Zaldivar and Ingram 1999, Almeida et al. 2007). High concentrations of ethanol, the product of fermentation itself, can affect cell membranes, cellular pH, and nutrient transport processes (Ingledew 2009, Ma and Liu 2010, Walker 2011). Thus, to achieve good ethanol yields from softwood derived sugars it is important to both increase the concentration of available sugars and find ways to deal with the inhibitors that will inevitably be associated with biomass derived sugars.

One strategy that we (Robinson et al. 2003) and other groups have used to increase the concentration of biomass derived sugars is to enzymatically hydrolyze and ferment whole slurries generated by pretreatment at high solids consistency. Such an approach, in addition to minimizing solid–liquid separation, enables the incorporation of the hemicellulose rich water soluble stream which can contain as much as one third of the sugars present in the original feedstock while enhancing the water use efficiency of the overall biomass-to-ethanol process. However, as mentioned earlier, hydrolysates generated by such a strategy are likely to provide a multi-stress environment with inhibitors and high sugar concentrations (high gravity) at the initial stages of fermentation and high ethanol concentrations at the latter stages. Consequently, fermentation conditions need to be optimized for efficient ethanol production under high gravity multi-stress conditions. While a number of studies have investigated the effects of model inhibitory compounds on fermentation in synthetic media (Palmqvist et al. 1999, Taherzadeh et al. 2000, Keating et al. 2006, Liu et al. 2004), only a few have looked at growth on industrially relevant lignocellulosic hydrolysates. While these earlier studies using model compounds proved to be very informative for a mechanistic understanding of inhibition, they provided little information on the likely synergistic interaction of the many inhibitory compounds that are prevalent in lignocellulosic hydrolysates.

In the work described here, a water soluble fraction (WSF) generated by steam pretreatment of Douglas-fir at high severity conditions was used to create a high gravity (high sugar concentration), multi-stress fermentation environment. The steam pretreatment of Douglas-fir, which is a softwood, results in a hemicellulose rich water soluble fraction (WSF) that is rich in hexoses with only small amounts of pentoses. Our objective was to establish a promising set of fermentation parameters for high gravity multi-stress fermentation of the hemicellulose rich water soluble fraction from steam treated Douglas-fir while developing a better understanding of the physiological/biochemical basis of yeast inhibitor tolerance. As will be detailed later, high cell density inoculations are needed if good ethanol productivity is to be achieved. Some yeast strains, especially those adapted to inhibitor-rich hydrolysates, were capable of both rapid conversion of furans and more efficient fermentation of the Douglas-fir WSF. High initial glucose concentrations also resulted in faster *in situ* detoxification of HMF. High cell density inoculation with minor supplemental nutrients could result in the successful high gravity multi-stress fermentation of softwood hydrolysates.

## Materials and methods

### Pretreatment

Steam pretreatment of Douglas-fir wood chips was performed as described previously (Ewanick et al. 2007). In brief, 75 g wood chips (by dry weight) were wetted overnight in 200 ml water in a plastic bag, at room temperature and subsequently impregnated with SO_2_ at 5% (w/w), based on the dry matter content of the raw material. The amount of SO_2_ was determined by weighing the bag before and after the addition of the gas. After 2 hours at room temperature, the treated chips were steam pretreated at 205°C for 10 min (severity factor, log R_o_ = 4.09) in a 2-L StakeTech II steam gun (Stake Technology, Norval, Canada). After pretreatment, the water soluble fraction (WSF) was separated from the water insoluble fraction (WIF) by filtration. Monomeric and oligomeric sugar concentrations of the WSF were determined by HPLC following standard protocols (Ewanick et al. 2007).

### *Saccharomyces cerevisiae* strains and preculture conditions

Strains LYCC 6391, LYCC 6492, LYCC 6961 and LYCC 6469 were provided by Lallemand, Inc. (Quebec, Canada). Tembec T1 and T2 strains of *S. cerevisiae* were obtained from Tembec Inc., (Temiscaming, Quebec, Canada). All strains were maintained on YPD agar plates containing 10 g/l yeast extract, 20 g/l peptone, 20 g/l glucose and 18 g/l agar at 4°C. For the precultures, yeast from a stock culture was propagated on YPD plates at 30°C for two days. A single yeast colony was transferred to 5 ml of YPD media in a sterile 50 ml Falcon tube and incubated overnight at 30°C in a rotary shaker at 150 rpm. About 1 ml of the preculture was subsequently transferred to a shake flask with 800 mL of YPD media and incubated until an OD of ≈ 0.8 was reached. The cells were harvested by centrifugation at 5000 rpm at ~21°C. Pellets were washed three times with sterile deionized water and re-suspended in sterile deionized water for use in fermentation trials.

### Fermentation

The pH of the Douglas-fir steam pretreatment WSF was adjusted to 5.5 with NaOH. Hereafter this is referred to as the original WSF (O-WSF). To create high gravity conditions, glucose was added to the O-WSF so that the final total monomeric sugar concentration (all five wood sugars) was 220 g/l. This is referred to as the glucose-added WSF (G-WSF). The fermentation trials were performed in 30 ml septic bottles with butyl-PFTE seals, with a working volume of 5 ml. Low and high cell density inoculations were conducted at 6 × 10^6^ cells/ml (OD_600_ ~ 0.5) and 150 × 10^6^ cells/ml (OD_600_ ~ 13), respectively. Reaction bottles were incubated in an orbital shaker for 48 hours at 30°C and 150 rpm. During the course of fermentation, 400 μl samples were taken at 0, 4, 8, 12, 24 and 48 h. The samples were centrifuged at 5 000 rpm for 5 min and the supernatant was stored at −20°C for further analysis. Prior to chemical analyses, all samples were filtered through 0.45 μm nylon filters.

### HPLC analysis

#### Determination of sugars

Sugars were measured on a Dionex (Sunnyvale, CA) HPLC (ICS-3000) equipped with an anion exchange column (Dionex CarboPac PA1). All sugars were detected via pulsed amperometry across a gold electrode with the use of deionized water at 1 mL/min as an eluent together with post-column addition of 200 mM NaOH. External standards of arabinose, galactose, glucose, xylose and mannose were used at six different levels to develop calibration curves for quantification of the sugar concentrations. Fucose was used as an internal standard.

#### Determination of inhibitors

Furfural and 5-hydroxymethyl furfural were determined using HPLC (ICS-500) fitted with an AS3500 autosampler, a UV detector and a GP40 gradient pump. The compounds were separated on an Aminex HPX-87H column (Biorad, Hercules, CA) at a temperature of 50°C using 5 mM H_2_SO_4_ at 0.6 ml/min as the eluent, and detected by UV absorbance. The concentration of total phenolics in the WSF was determined using the Folin-Ciocalteu reagent (Sigma) Singleton and Rossi ( 1965, Robinson 2003). For each sample 40 μl were diluted up to 1 ml with nanopure water. To 100 μl of diluted sample, 250 μl of Folin-Ciocalteau reagent were added. After 5 min, the reaction was stopped by the addition of 750 μl of 20% (w/v) Na_2_CO_3_ and the final volume was brought up to 5 ml with nanopure water. The reaction mixtures were incubated for 2 hours at 22°C with constant mixing on a magnetic stir-plate. The absorbance of each reaction was measured spectrophotometrically at 760 nm. Vanilin was used as an external standard at six levels. Reactions were done in duplicate and the average value was reported.

#### GC-FID

Ethanol quantification was done by gas chromatography on a 5890 Series II chromatograph with a 6890 autoinjector with a splitless injector system and a flame ionisation detector (Hewlett Packard, Palo Alto, CA). Helium was used as the carrier gas (20 ml/min) for injection into a HP-Innowax analytical column (15 m × 0.53 mm). In brief, the temperatures of the injection unit and flame ionization detector (FID) were set at 175°C and 250°C, respectively. The oven was heated to 45°C for 2.5 minutes and the temperature was raised to 110°C at a rate of 20°C/min and later held at 110°C for 2 minutes. Standards and samples were supplemented with 1-butanol as the internal standard.

## Results

As detailed in the Materials and Methods section, the water soluble fraction (WSF) obtained after acid catalysed steam pretreatment of Douglas-fir wood chips was rich in hemicellulose derived sugars with a final sugar concentration of 17.9 g/l glucose, 9.4 g/l mannose, 2.3 g/l galactose, 2.1 g/l xylose and 1.0 g/l arabinose detected. There were also low concentrations of HMF (2.4 g/l) and furfural (0.5 g/l) detected even though the severity of pretreatment was chosen to minimise sugar degradation while maximising hemicellulose solubilisation and sugar recovery in a monomeric form. The WSF also contained 3.0 g/l acetic acid and 1.7 g/l total soluble phenolics. These concentrations are in agreement with the sugar and inhibitor concentrations typically found in the WSF after steam treatment of softwoods (Almeida et al. 2007, Ewanick et al. 2007, Kumar et al. 2010).

### Fermentation of the WSF obtained after steam treatment of Douglas-fir

**Figure 1:**
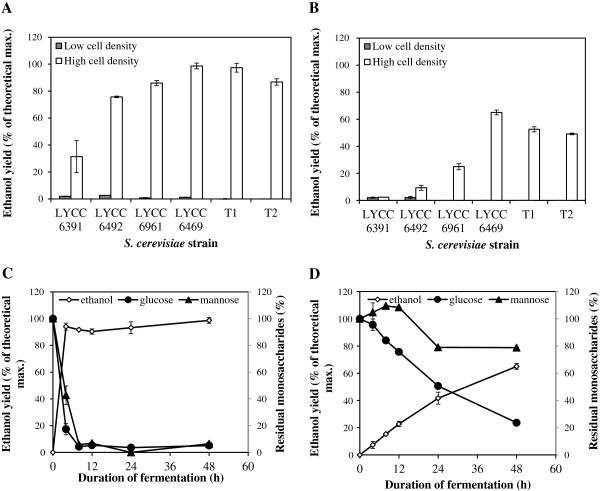
**Ethanol production by the tested *S. cerevisiae *strains from the steam pretreatment water soluble fraction (WSF) at low and high cell density inoculation A: O-WSF: without glucose supplementation B: G-WSF: glucose added. C**: Time-course of ethanol production and monosaccharide consumption from O-WSF by Lallemand LYCC 6469 at high cell density. **D**: Time-course of ethanol production and monosaccharide consumption from G-WSF by Lallemand LYCC 6469 at high cell density.

When inoculated with a relatively low cell density of 6 × 10^6^ cells/ml, none of the tested *S. cerevisiae* strains produced appreciable amounts of ethanol when grown on the original WSF (O-WSF) with no glucose supplementation (Figure 1A). However, when a high cell density of 150 × 10^6^ cells/ml was used, most strains resulted in >70% ethanol yield after 48 h, with the Lallemand LYCC 6469 and Tembec T1 stains resulting in a ~98% yield (percent ethanol yields were calculated based on total available monomeric hexoses assuming a maximum yield of 0.51 g ethanol per gram of hexose). These two strains consumed all of the glucose and mannose after 8 h and 4 h, respectively (Figure 1C, only data for LYCC 6469 are shown). However, with the other strains, such as LYCC 6391, substantial amounts of all monomeric sugars (50-100%) remained, even after 48 h of incubation (data not shown).

When glucose was added to the WSF, low cell density fermentations were unsuccessful. In this case, strain LYCC 6469 when added at a high cell density performed the best, resulting in a 65% ethanol yield (based on total available monomeric hexose) after 48 h (Figure 1B and D). However, this represented a 34% decrease in ethanol yield when compared to the WSF without supplemental glucose addition (Figure 1C) although, due to the addition of the glucose, a final concentration of ~72 g/l ethanol could be achieved. The Tembec T1 and T2 strains showed comparable ethanol concentrations of ~62 g/l ethanol with an approximately 50% yield. The highest volumetric productivity values (Q_EtOH (0–4 h)_, 1–2 g/l/h) were achieved with the Tembec and LYCC 6469 strains. Under the multi-stress conditions of high gravity fermentations, none of the hexoses were completely consumed by any of the yeast strains. The LYCC 6469 strain utilized about 75% and 25% of available glucose and mannose, respectively (Figure 1D), while the Tembec T1 and T2 reached ~65% glucose and ~45% mannose utilization. Galactose was barely metabolized by any of the evaluated strains (data not shown).

It was apparent that, under high gravity multi-stress conditions, all of the yeasts struggled to attain efficient ethanol production and that supplemental nutrients might be required if ethanol yields of >80% are to be achieved. When a cocktail of 0.5 g/l NH_4_H_2_PO_4_, 0.025 g/l MgSO_4_.7H_2_O, and 1.0 g/l yeast extract was added to the glucose supplemented WSF (G-WSF) a ~25% increase in yeast growth rate (Additional file 1: Figure S1) and an increased ethanol yield of 77%, based on the available hexoses, were obtained after growth of the LYCC 6469 strain at high cell density.

### *In situ* detoxification of fermentation inhibitors in the Douglas-fir WSF

**Figure 2:**
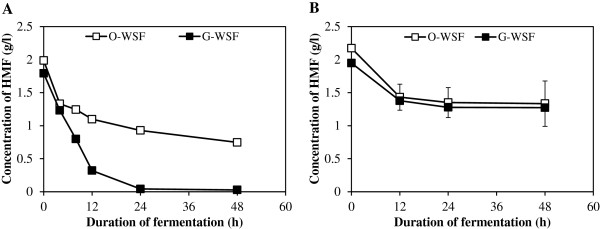
***In situ *conversion of HMF in the original WSF (O-WSF) and the glucose-added WSF (G-WSF) by Lallemand LYCC 6469 (A) and 6391 (B) during high cell density fermentation.**

In previous work, we observed (Liu, 2010) that moderate levels of glucose (100 g/l) improved the fermentability of softwood hydrolysates. To try to better understand the physiological basis of this phenomenon, furan metabolism by different strains under high gravity and low gravity (low sugar) conditions was next investigated. Data shown in Figures 1B and 2 clearly demonstrate an association between HMF disappearance and strain performance. The tested strains could be broadly categorized into three groups based on ethanol production from high gravity fermentation of the Douglas-fir WSF. The best performers included LYCC 6469 and Tembec T1 and T2, while the poorest comprised LYCC 6391 and LYCC 6492. The performance of LYCC 6961 was moderate in comparison (Figure 1B). At high cell density, more than 98% of the HMF in the glucose supplemented WSF had been metabolised by LYCC 6469 after 24 h. However, without added glucose, there was only a 62% reduction in HMF after 48 h, i.e. glucose supplementation enabled a 2-fold increase (during the first 12 h of fermentation) in the rate of HMF degradation (Figure 2). Comparable results were obtained with Tembec T1 and T2 (data not shown). LYCC 6961 converted 74% of HMF in the glucose supplemented WSF compared to 37% in the original WSF (Additional file 2: Figure S2). The poorest ethanol producers, LYCC 6391 and LYCC 6492 consumed only 35% of the HMF after 48 h growth in the WSF, either with or without glucose supplementation. At low cell densities, there was very little change in the HMF concentration after 48 h with only a few strains, including T1 and T2, resulting in a 6-7% decrease in the HMF concentration. This seemed to indicate that those strains that were better able to reduce HMF were also able to achieve higher ethanol yields at higher productivity. In addition, these superior strains demonstrated better HMF reduction rates when supplemental glucose was added.

**Figure 3:**
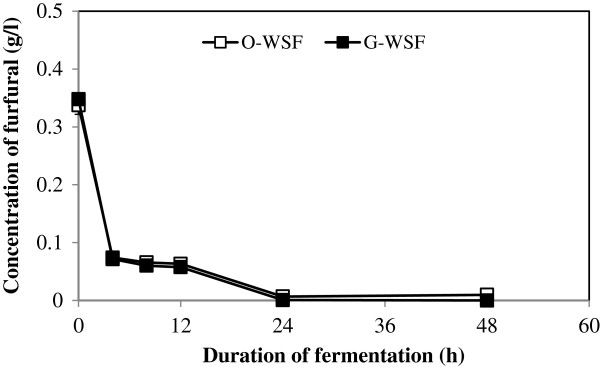
***In situ *conversion of furfural in the original WSF (O-WSF) and the glucose-added WSF (G-WSF) by Tembec T2 during high cell density fermentation.**

In contrast to the trends observed with HMF metabolism, all of the strains except LYCC 6391, at high cell density, converted 97-100% furfural from glucose supplemented and un-supplemented WSFs within 24 h (Figure 3). Even at low cell density, Tembec T1 and T2 could reduce the furfural concentration by 15-30% after 48 h of fermentation from the original and glucose-added WSFs. Added glucose did not result in any faster removal of furfural from the Douglas-fir derived WSF.

**Figure 4:**
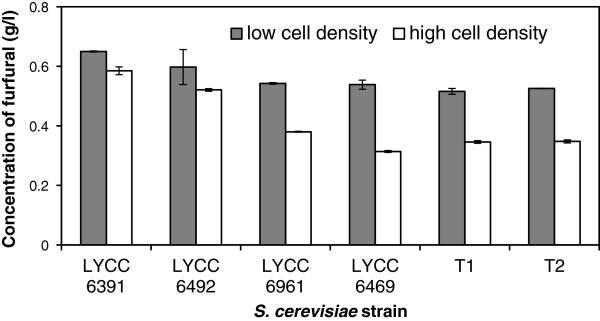
Effect of cell density on the initial furfural concentration in the glucose-added WSF (G-WSF).

It was clear that high cell density inoculations led to an almost immediate decrease in the furfural concentration (Figure 4). When the concentrations of furfural in the samples collected immediately after mixing yeast with the WSF were assessed, Tembec T1, T2, LYCC 6469 and LYCC 6961 strains at high cell density resulted in 30-34% reduction in furfural concentration of the glucose-added WSF (G-WSF). Use of the high cell density incubation approach with strains LYCC 6391 and LYCC 6492 resulted in a 10-13% reduction in the furfural found in the G-WSF. These observations cannot be attributed to volume changes because water was used to compensate for differences in low and high cell density inoculations. In the case of HMF, high cell density inoculations only resulted in small (~6%) reductions in the initial concentrations (data not shown).

## Discussion

For biomass derived ethanol to ever compete with traditional sugar or starch based commercial processes it would be extremely beneficial if an ethanol titer of at least 4% (w/v) with >80% yield could be achieved. One promising strategy to try to realize these goals is to process pretreated biomass slurries (combining the hemicellulose and cellulose derived sugars of the biomass feedstock) at high solid consistencies (i.e. high sugar concentrations). However, process parameters that incorporate ways to provide good ethanol production under conditions of high sugar, high inhibitors, high ethanol and low additional nutrient concentrations first need to be established. In earlier work (Schwald et al. 1988, Kumar et al. 2010) it was shown that optimised steam pretreatment conditions not only “opened-up” the cellulosic component of biomass substrates so that enzymatic hydrolysis could be enhanced, under the proper conditions it also provided the recovery of most of the hemicellulose sugars in the water soluble fraction (WSF). However, the hemicellulose rich water soluble fraction also contained most of the inhibitory material, both naturally occurring and process derived, that any fermentative organism would have to deal with (Robinson et al. 2003, Almeida et al. 2007). As noted in earlier work (Robinson et al., 2003) it is technically very difficult to produce biomass derived sugar at the high, ≥ 200 g/l, concentrations typically used in commercial sugar- and starch-based ethanol processes (Bai et al., 2008, Ingledew 2009, Walker 2011), primarily because of the high solid (pulp) consistencies (~40% w/w) that would be required. The few studies that have reported sugar concentrations in excess of 200 g/l used delignified lignocellulosic substrates and high doses of cellulolytic enzymes (Yang et al. 2010, Zhang et al. 2009). Therefore, to try and at least simulate high gravity (high sugar) concentrations in the presence of most of the anticipated inhibitory materials, supplemental glucose was added to the hemicellulose rich water soluble stream obtained after steam pretreatment of Douglas-fir wood chips. As described earlier we investigated three process parameters pertinent to the high gravity fermentation of the sugar present in glucose supplemented or unsupplemented steam pretreated water soluble fractions. These included the nature of the yeast strain used, the inoculation cell density and the requirement for any additional nutrients.

The strains Lallemand LYCC 6469, Tembec T1, and T2 were best able to ferment the wood derived sugars. The Tembec T1 and T2 strains are natural isolates from spent sulfite liquor (SSL) which is the residual liquid stream obtained after the ammonium bisulfite digestion of wood (Boussaid et al. 2001, Helle et al. 2003). In addition to its high osmotic strength, SSL is rich in numerous fermentation inhibitors including acetic acid, furfural, HMF, lignosulfonates, and sulfate (Helle et al. 2004). Through long-term exposure to SSL, the T1 and T2 strains have been adapted for survival and ethanol production under adverse environmental conditions. Our earlier work had shown that the T1 and T2 strains could ferment most of the sugars present in softwood hydrolysates (Liu 2010) while other SSL-adapted strains of *S. cerevisiae* (TMB3000 and USM21) have also been shown to be better able to ferment lignocellulosic hydrolysates to ethanol (Laadan et al. 2008, Nilsson et al. 2005).

However, when using a low cell density inoculation, even the more robust yeast strains did not grow or ferment the sugars well. In contrast, as has been shown by other workers, (Chung and Lee 1985, Boyer et al. 1992, Palmqvist et al. 1998) high cell density inoculations resulted in a significant and sometimes rapid reduction in some of the recognized fermentation inhibitors. Another beneficial effect is that high cell concentrations tend to down-regulate yeast cell growth, resulting in reduced carbon drain for growth. As a consequence, more carbon can be used for ethanol production (Melzoch et al. 1991, Palmqvist et al. 1998) while a higher cell density, due to the increased number of “working cells”, enables higher volumetric ethanol productivity (Borzani et al., 1993, Navaro 1994, Palmqvist et al. 1998, Cardona and Sanchez 2008, Walker 2011). In related work, continuous fermentation using spruce enzymatic hydrolysate showed 4.6 times higher productivity values under high cell density conditions (Palmqvist et al. 1998). Similarly, when the hydrolysate from steam pretreated sweet sorghum bagasse was fermented, increasing the cell density from 1 g/l to 3 g/l led to a >3-fold increase in productivity (Shen et al. 2012). Productivity improvements have also been reported when using molasses and sugarcane bagasse hydrolysate (Palmqvist et al. 1998, Canilha et al., 2010, Canihla et al., 2012, Nofemele et al., 2012).

The work reported here confirmed the earlier observation by Liu (2010) that glucose supplementation improved the fermentability of the sugars present in the water soluble fractions of steam pretreated softwoods. It has been suggested that, in the presence of high glucose concentrations, ATP can be more readily regenerated to meet the increased maintenance energy demand under inhibitor stress (Helle and Duff 2004). Transcriptome analyses indicated that HMF tolerant *S. cerevisiae* strains reprogram metabolic pathways to maintain energy metabolism and redox balance by creating a short cut to the TCA cycle to enhance ATP and NADPH synthesis. As a result, glycolysis is repressed and glucose metabolism is rewired to continue through the pentose phosphate pathway, the main route to NADPH regeneration in yeast (Ma and Liu 2010, Liu 2011). NADPH is necessary for the reduction of HMF into HMF alcohol, while ATP is possibly used, among other processes, for pumping toxic products generated by inhibitor damage out of the cells (Liu 2011). It is also possible that the beneficial glucose effect on HMF removal may be an indirect result of changes in gene expression in response to osmotic stress caused by high sugar. Certain stress factors are known to cause changes in expression that enable tolerance to other unrelated stresses (Liu 2011). In the case of the high gravity multi-stress fermentations studied here, the relatively high sugar concentrations used might have induced further expression of the reductases capable of detoxifying HMF.

As well as looking at the influence of high sugar concentrations and inhibitors on the fermentation of wood derived sugars, supplementation of nutrients cocktail, containing 0.5 g/l NH_4_H_2_PO_4_, 0.025 g/l MgSO_4_.7H_2_O, and 1.0 g/l yeast extract could significantly improve the ethanol yields of the sugar supplemented water soluble fraction (WSF). Lignocellulosic hydrolysates generally lack some of the key nutrients that are needed for optimal yeast growth and stress tolerance. Other workers have successfully used this cocktail to enhance yeast growth and ethanol production when grown on the sugars present in un-detoxified softwood slurries (Rudolf et al. 2007, Bertilsson et al. 2009, Olofsson et al. 2010).

In conclusion, several important process parameters were better defined to help achieve effective high gravity fermentation of softwood derived wood sugars. When the Lallemand LYCC 6469, Tembec T1, and T2 yeast strains were used at a high cell density of at least 150 × 10^6^ cells/ml both the furfural and HMF present in the glucose supplemented, hemicellulose rich water soluble fraction were quickly removed. An ethanol yield of 77% could be achieved after 48 h when strain LYCC 6469 was grown at high cell density with nutrient supplementation. As the water soluble fraction of steam pretreated biomass is known to contain most of the inhibitory material present in the original and processed biomass, it is likely that a similar approach of using inhibitor adapted yeast at high cell density can be effectively used to ferment high consistency slurries of softwood derived cellulose and hemicellulose sugars.

## Competing interests

The authors declare that they have no competing interests.

## Supplementary Material

Additional file 1: Figure S1 Effect of nutrient supplementation on the growth of Lallemand LYCC 6469 during high cell density fermentations of the glucose-supplemented water soluble fraction (G-WSF). Click here for file

Additional file 2: Figure S2 *In situ* conversion of HMF in the original (O-WSF) and the glucose-added water soluble fraction (G-WSF) by Lallemand LYCC 6961 during high cell density fermentation. Click here for file
