# Supplementary material for: High gravity and high cell density mitigate some of the fermentation inhibitory effects of softwood hydrolysates
Source: AMB Express. 2013 Feb 14;3:15. doi: 10.1186/2191-0855-3-15 (PMC3576264; doi:10.1186/2191-0855-3-15)
Supplement: Supplementary file 1 [file 2191-0855-3-15-S1.doc]

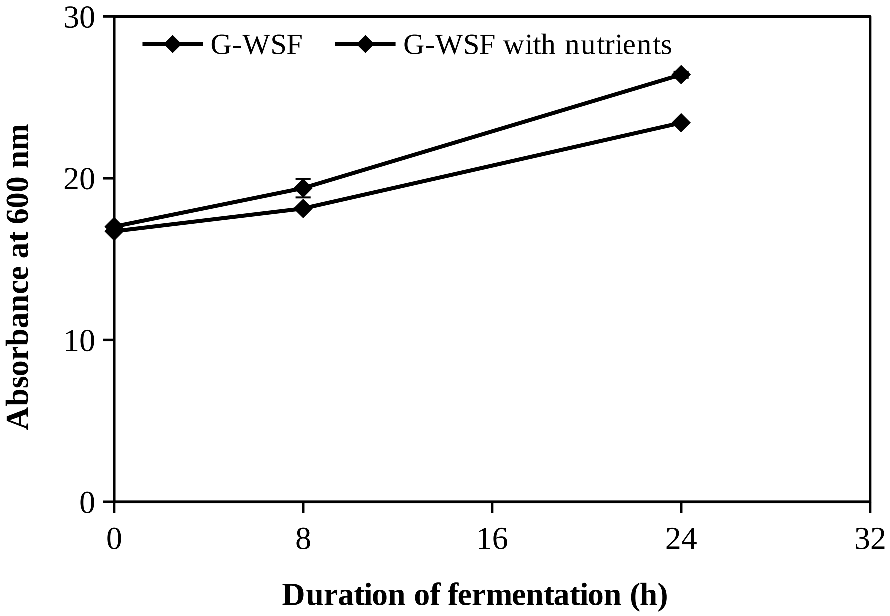

G-WSF
G-WSF with nutrients
Absorbance at 600 nm
Duration of fermentation (h)
0
10
20
30
0
8
16
24
32
