# Supplementary material for: High gravity and high cell density mitigate some of the fermentation inhibitory effects of softwood hydrolysates
Source: AMB Express. 2013 Feb 14;3:15. doi: 10.1186/2191-0855-3-15 (PMC3576264; doi:10.1186/2191-0855-3-15)
Supplement: Supplementary file 2 [file 2191-0855-3-15-S2.doc]

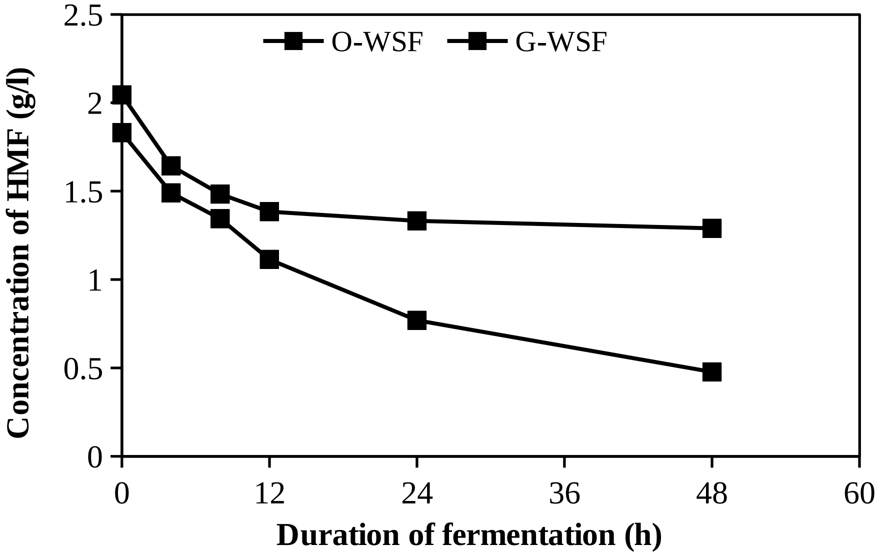

O-WSF
G-WSF
Concentration of HMF (g/l)
Duration of fermentation (h)
2.5
2
1.5
1
0.5
0
0
12
24
36
48
60
